# RORA alleviates LPS-induced apoptosis of renal epithelial cells by promoting PGC-1α transcription

**DOI:** 10.1007/s10157-022-02184-2

**Published:** 2022-02-23

**Authors:** Dayong Li, Guanlan Liu, Yundou Wu

**Affiliations:** Department of Nephrology, The First Hospital of Changsha, No. 311 Yingpan Road, Changsha, 410005 Hunan People’s Republic of China

**Keywords:** RORA, PGC-1α, Lipopolysaccharide, Renal epithelial cells, Apoptosis

## Abstract

**Objective:**

To explore the effect of RORA on LPS-induced renal epithelial cell apoptosis and the underlying mechanism.

**Methods:**

LPS-treated HK-2 cells were established as a cellular model of acute kidney injury. The expression of RORA or/and PGC-1α in LPS-induced HK-2 cells was altered by transfection. qRT-PCR and Western blotting were used to detect the expression changes of RORA and PGC-1α. ELISA was performed to detect the expression of IL-1β and IL-6 and the activity of caspase-3. Western blotting was applied for visualization of cleaved caspase-3. CCK-8 and flow cytometry were used to assess cell proliferation and apoptosis. Dual-luciferase reporter and ChIP-qPCR were utilized to verify the binding of RORA to PGC-1α promoter.

**Results:**

LPS treatment decreased the expression of RORA and PGC-1α and increased that of cleaved caspase-3 in HK-2 cells. Also, LPS treatment inhibited HK-2 cell proliferation and promoted HK-2 cell apoptosis and secretion of IL-1β and IL-6. Overexpression of RORA or PGC-1α eliminated the adverse effects of LPS treatment in HK-2 cells. RORA drove the transcription of PGC-1α by binding PGC-1α promoter. Knockdown of PGC-1α offset the reduction in HK-2 cell injury caused by overexpression of RORA.

**Conclusion:**

RORA reduces LPS-induced apoptosis of renal epithelial cells by promoting PGC-1α transcription.

## Introduction

Acute kidney injury (AKI) is a syndrome characterized by a sudden increase in serum creatinine or/and a decrease in urine output, which occurs in 10–15% of all hospitalized patients, especially in the intensive care unit [1]. Current management of AKI includes optimization of hemodynamics and discontinuation of nephrotoxic medications in general cases and renal replacement therapy in acute settings [2]. By far, there are no targeted pharmacotherapies approved for the treatment of AKI. A wide range of pathophysiological events occur particularly in proximal tubule cells after AKI and subsequently lead to cell deaths [3]. Molecules involved in renal cell deaths such as apoptosis can be utilized as targets for the diagnosis and treatment of AKI.

Retinoic acid receptor-related orphan receptor (ROR) alpha (RORA) gene, located at human chromosomal band 15q22.2, is frequently activated in response to different cellular stresses and encodes an evolutionarily related transcription factor ROR [4]. RORA plays a critical role in regulation of inflammation and its polymorphisms have recently been reported to have associations with susceptibility to migraine, pediatric asthma and multiple sclerosis [5–7]. A study by Cai and colleagues showed that RORA was downregulated in the kidney of mice after ischemia/reperfusion (I/R) injury and that a deficiency of RORA contributed to tubular epithelial cell apoptosis and consequently led to renal dysfunction [8], but the downstream targets of RORA in regulating renal cell apoptosis remained unclear.

Peroxisome proliferator-activated receptor gamma coactivator 1 alpha (PGC-1α) is a master regulator of mitochondrial biogenesis and as a coactivator for multiple transcription factors and proteins, it controls metabolism and various tissue-specific processes [9]. PGC-1α is downregulated under inflammatory conditions, which results in decreased expression of mitochondrial antioxidant genes and disturbs redox homeostasis [10]. Low levels of PGC-1α have been observed in both preclinical and experimental AKI; and several therapies have been shown to protect the kidney in animal models by increasing the activity of PGC-1α [11], 12, 13.

Based on the existing evidence, RORA and PGC-1α are two nephroprotective proteins exhibiting low expression in AKI. As RORA is a transcription factor, we hypothesized that RORA regulated the transcription of PGC-1α to reduce renal epithelial cell apoptosis after AKI.

## Materials and methods

### Cell culture and LPS treatment

Kidney epithelial cell line HK-2 was purchased from the Cell Bank of the Chinese Academy of Sciences (Shanghai, China) and authenticated. HK-2 cells were cultured in DMEM (Gibco, Grand Island, NY, USA) supplemented with 10% fetal bovine serum (FBS; Thermo Fisher Scientific, Waltham, MA, USA) and antibiotics (Gibco) at 37 °C with 5% CO_2_. A LPS-induced renal epithelial cell apoptosis model was established based on a previous study. Briefly, HK-2 cells were incubated with 0, 1, 2, 5, or 10 μg/mL LPS (Sigma-Aldrich, St. Louis, MO, USA) for 8 h before subsequent experiments.

### Cell transfection

RORA overexpression vector (OE-RORA) and its negative control (OE-NC), PGC-1α overexpression vector (OE-PGC-1α) and its negative control (OE-NC), PGC-1α inhibition vector (sh-PGC-1α) and its negative control (sh-NC) were purchased from GenePharma (Shanghai, China). OE-RORA, OE-NC, and OE-PGC-1α were delivered at a concentration of 5 μg/mL, while sh-PGC-1α and sh-NC were delivered at 5 μL/mL. The duration of OE-RORA or OE-PGC-1α transfection lasted for 72 h before LPS treatment, while that of sh-PGC-1α transfection lasted for 48 h before LPS treatment. Briefly, cells were seeded into a 6-well plate 24 h before transfection. Transfection vectors were diluted with 250 μL of serum-free Opti-MEM (51,985,042, Gibco, Grand Island, NY, USA) and incubated at room temperature for 5 min. Lipofectamine 2000 (5 μL; 11,668–019, Invitrogen, Carlsbad, CA, USA) was diluted with 250 μL of serum-free Opti-MEM and incubated at room temperature for 5 min. The two solutions were mixed and incubated at room temperature for 20 min before being added into the plate. Then, the cells with 40–70% confluency were incubated with the plasmids at 37 °C with 5% CO_2_ for 6–8 h, after which the culture medium was replaced with complete medium.

### Western blotting

Cells were lysed with RIPA buffer (Beyotime, Shanghai, China) on ice for 15 min and centrifuged at 13,000*g* for 5 min, followed by measurement of the concentrations of protein samples using a BCA kit (Beyotime). Protein samples were added into loading buffer and denatured in boiling water for 10 min. The loading volume of each sample was calculated using a constant amount. The samples were electrophoresed on gels at 80 V for 30 min and after bromophenol blue entered the separation gel, the electrophoresis was run at 120 V for 90 min. Separated proteins of each group were transferred onto a PVDF membrane in an ice bath (250 mA, 100 min). The membrane was washed three times (1–2 min each time) and placed in blocking solution for 2 h before incubation with antibodies against RORA (1:1000, ab70061, Abcam, Cambridge, MA, USA), PGC-1α (1:1000, ab54481, Abcam), caspase-3 (1:5000, ab32351, Abacm) and cleaved caspase-3 (1:500, ab2302, Abacm) at 4 °C overnight. The membrane was incubated with secondary antibody (HRP-labeled goat anti-rabbit IgG; 1:1000, A0208, Beyotime) for 2 h at room temperature. The membrane was washed with TBST for 3 × 10 min before and after incubation with secondary antibody. Finally, the membrane was treated with ECL color developing solution (P0018FS, Beyotime) and images were captured by a chemiluminescence imaging system (Bio-rad, Hercules, CA, USA). Each experiment was repeated 3 times.

### Quantitative real-time PCR (qRT-PCR)

TRIzol reagent was used for extraction of total RNA from cells and a reverse transcription kit (Takara, Tokyo, Japan) for synthesis of cDNA. SYBR Green Mix (Takara) was used for qRT-PCR that was performed on the Applied Biosystems 7300 Real-Time PCR System (ABI, Waltham, MA, USA). Each sample had three duplicates. The ${2}^{{ - \Delta \Delta C_{{\text{t}}} }}$ method [14] was adopted for data analysis. ΔΔ*C*_t_ = (*C*_t target gene_ – *C*_t internal reference_)_experimental group_ – (*C*_t target gene_ – *C*_t internal reference_)_control group_. GAPDH was used as a reference gene. See Table 1 for the information of used primers.

**Table 1:** Primer information

Name	Sequences (5′ to 3′)
RORA-F	GAGCCATCTGTCTGATCACC
RORA-R	CTCAGGGAGCTACAGGTTGA
PGC-1α-F	ATGAAGGGTACTTTTCTGCCC
PGC-1α-R	ACCACTTGAGTCCACCCAGA
GAPDH-F	CACCCACTCCTCCACCTTTG
GAPDH-R	CCACCACCCTGTTGCTGTAG

F, forward primer; R, reverse primer

### CCK-8 for assessing cell proliferation

Cells were seeded into a 96-well plate with 3 duplicates for each sample and then each well was added with 10 μL of CCK-8 solution. The plate was placed in a CO_2_ incubator for 0 h, 24 h, 48 h or 72 h and then transferred to a microplate reader (Model 680, Bio-Rad, Hercules, CA, USA) for measurement of absorbance at 450 nm. Each experiment was repeated 3 times.

### ELISA

The expression levels of inflammatory factors IL-1β and IL-6 were detected with ELISA kits (IL-1β: ab46052, Abacm, Cambridge, MA, USA; IL-6: ab222503, Abacm). First, 100 μL of test sample or standard solution was added into the test well and incubated at 37 °C for 90 min. Then, the sample was incubated with specific antibody for 60 min and avidin–biotin-peroxidase complex for 30 min. After treatment with TMB color developing reagent for 20–25 min, the sample was subjected to measurement of absorbance at 450 nm.

The activity of apoptotic factor caspase-3 was detected using a caspase-3 detection kit (ab252897; Abcam). Briefly, cells were lysed and experiments were performed following the user’s manual. Absorbance was measured at 450 nm by a microplate reader. Each experiment was repeated 3 times.

### Flow cytometry

Cells were trypsinized and collected by centrifugation. The cells were resuspended in binding buffer and incubated with Annexin V-FITC at room temperature for 15 min. The apoptosis of the cells was examined by a flow cytometer. Each experiment was repeated 3 times.

### Dual-luciferase reporter assay

Jaspar (http://jaspar.genereg.net/) predicted the binding sites between RORA and PGC-1α. Wild sequence (wt-PGC-1α) and mutant sequence (mut-PGC-1α) of the binding sites on PGC-1α were designed and synthesized according to the predicted results. Wt-PGC-1α or mut-PGC-1α was inserted into pGL3-Basic vector and then delivered together with OE-RORA or OE-NC into HEK293T cells. A dual-luciferase reporter kit (Promega, Madison, WI, USA) and a luminometer (Turner BioSystems, Sunnyvale, CA, USA) were used to detect the luminescence activity of each group.

### Chromatin immunoprecipitation (ChIP)

DNA–protein crosslinks were produced by formaldehyde fixation (10 min). Chromatin was fragmented by sonication (15 times, 10 s each time with an interval of 10 s). The cell lysate was centrifuged at 12,000*g* (4 °C) for 10 min. Supernatant was collected and divided into two parts. One part was incubated with anti-RORA (sc-518081, Santa Cruz Biotechnology, Santa Cruz, CA, USA) at 4 °C overnight and the other with anti-IgG (ab172730, Abcam, Cambridge, MA, USA). DNA–protein complexes were precipitated by Protein Agarose/Sepharose and collected by a spin at 12,000*g* for 5 min. Non-specific binding complexes were washed off. After de-crosslinking at 65 °C overnight, DNA fragments were extracted and purified with phenol/chloroform. PGC-1α-specific primers (forward: 5′-AAAAGGGTTATCTGGGGGCG-3′; reverse: 5′-GGAATGGTCCACAAAAGGGC-3′) were used for qPCR to detect the binding of RORA and PGC-1α.

### Statistical analysis

Data were statistically analyzed by GraphPad Prism 7 and all expressed as mean ± standard deviation. *T* test was used for comparisons between two groups and one-way analysis of variance for multiple groups. Tukey’s test was applied for post hoc multiple comparisons. A difference with *P* < 0.05 was considered statistically significant.

## Results

### Overexpression of RORA reduces LPS-induced renal epithelial cell apoptosis

**Fig. 1:**
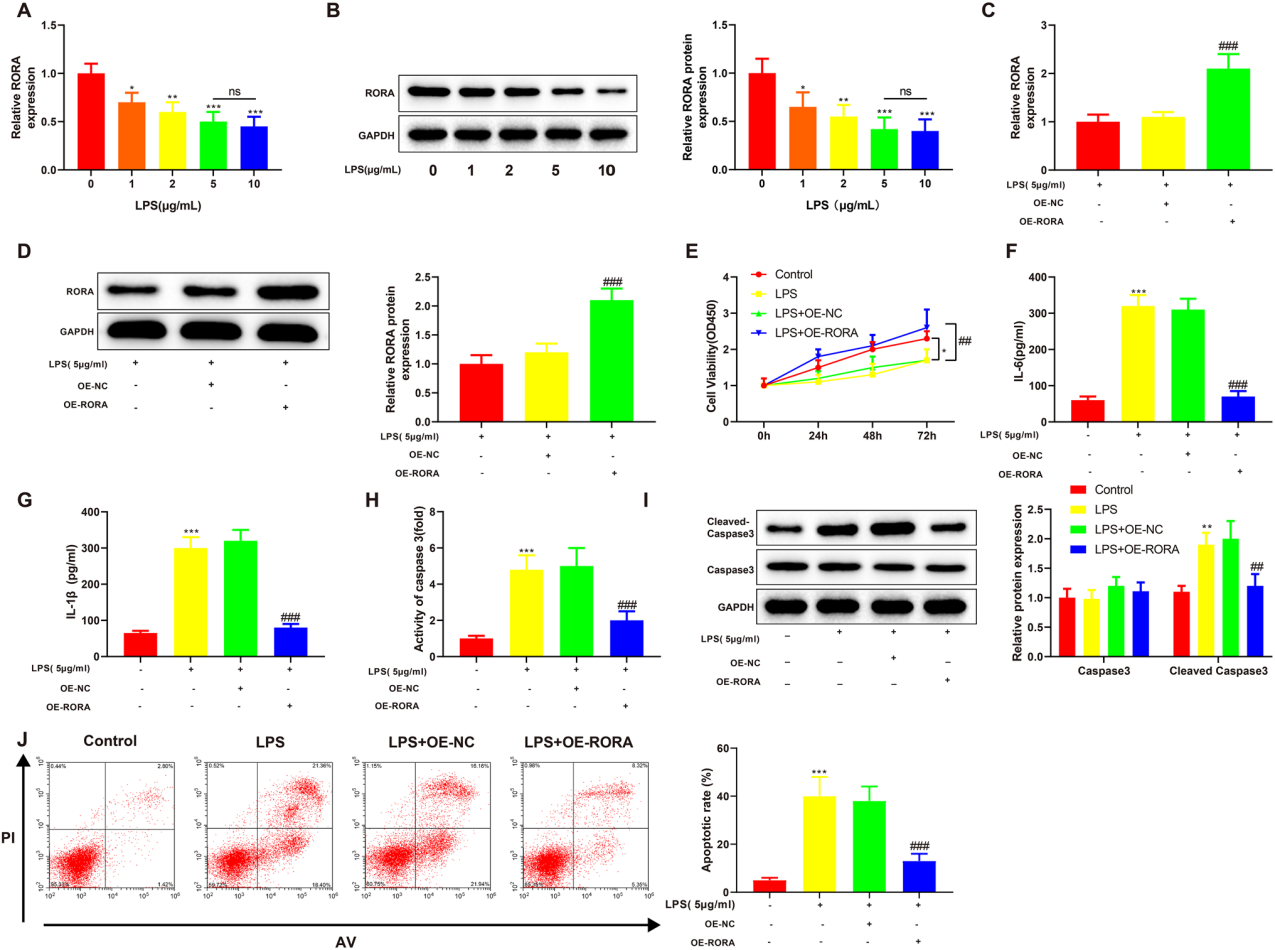
Overexpression of RORA reduces LPS-induced renal epithelial cell apoptosis. qRT-PCR (**A**) and Western blotting (**B**) were used to detect the expression of RORA mRNA and protein in HK-2 cells treated with different concentrations of LPS (0, 1, 2, 5 and 10 μg/mL). qRT-PCR (**C**) and Western blotting (**D**) were used for analyzing the transfection efficiency of OE-RORA in LPS-treated HK-2 cells. **E** CCK-8 was used for evaluating the proliferation of LPS-treated HK-2 cells before and after OE-RORA transfection. ELISA was performed to detect the expression of IL-6 (**F**) and IL-1β (**G**) in the supernatant of LPS-treated HK-2 cells before and after OE-RORA transfection. **H** ELISA was performed to detect the activity of caspase-3 in LPS-treated HK-2 cells before and after OE-RORA transfection. **I** Western blotting was used to detect caspase-3 and cleaved caspase-3 in LPS-treated HK-2 cells before and after OE-RORA transfection. **J** Flow cytometry was applied for analyzing the apoptosis of LPS-treated HK-2 cells before and after OE-RORA transfection. Data were expressed as mean ± standard deviation and each experiment was repeated 3 times. *P < 0.05, **P < 0.01 and ***P < 0.001, compared with 0 μg/mL or control group. ^##^*P* < 0.01 and ^###^*P* < 0.001, compared with LPS + OE-NC group. Abbreviations: RORA, retinoic acid receptor-related orphan receptor alpha; LPS, lipopolysaccharide; qRT-PCR, quantitative real-time polymerase chain reaction; OE-RORA, RORA overexpression vector; OE-NC, negative control vector; CCK-8, cell counting kit-8; ELISA, enzyme-linked immunosorbent assay; IL, interleukin

To study the mechanism of RORA on AKI, we established AKI cell models by treating human renal epithelial HK-2 cells with different concentrations of LPS. First, the expression of RORA in the HK-2 cells was detected. The results of qRT-PCR and Western blotting showed that LPS treatment (1, 2, 5 or 10 μg/mL) significantly reduced the expression levels of RORA mRNA and protein in HK-2 cells (Fig. 1A, B, ***P < 0.001). Treatment with 5 μg/mL LPS and 10 μg/mL LPS resulted in no significant difference in the expression levels of RORA mRNA and protein in HK-2 cells. Therefore, we chose 5 μg/mL LPS to be used in the following experiments.

Next, we delivered RORA overexpression vector (OE-RORA) or its negative control (OE-NC) into LPS-treated HK-2 cells. The results of qRT-PCR and Western blotting showed that OE-RORA transfection significantly increased the expression levels of RORA mRNA and protein in LPS-treated HK-2 cells (Fig. 1C, D, ^###^*P* < 0.001). We tested the viability of HK-2 cells using CCK-8. The proliferative ability of HK-2 cells was impaired by LPS treatment (Fig. 1E, *P < 0.05) and then rescued by OE-RORA transfection (Fig. 1E, ^##^*P* < 0.01). ELISA was performed to detect the expression of inflammatory factors IL-1β and IL-6 in cell supernatant and the activity of apoptosis factor caspase-3 in cells. LPS treatment increased the expression of IL-1β and IL-6 and the activity of caspase-3 (Fig. 1F–H, ***P < 0.001), which was counteracted by OE-RORA transfection (Fig. 1F–H, ^###^*P* < 0.001). In addition, the Western blots showed there was an increase in the expression of cleaved caspase-3 in HK-2 cells after LPS treatment (Fig. 1I, **P < 0.001) and a decrease in the expression of cleaved caspase-3 in LPS-treated HK-2 cells after OE-RORA transfection (Fig. 1I, ^##^*P* < 0.01). The flow cytometry analysis showed that LPS treatment promoted the apoptosis of HK-2 cells (Fig. 1J, ***P < 0.001) and that OE-RORA transfection significantly reduced the apoptosis of LPS-treated HK-2 cells (Fig. 1J, ^###^*P* < 0.001). Collectively, these data indicated that overexpression of RORA reduced LPS-induced apoptosis of HK-2 cells.

### RORA binds PGC-1α promoter and promotes PGC-1α transcription

**Fig. 2:**
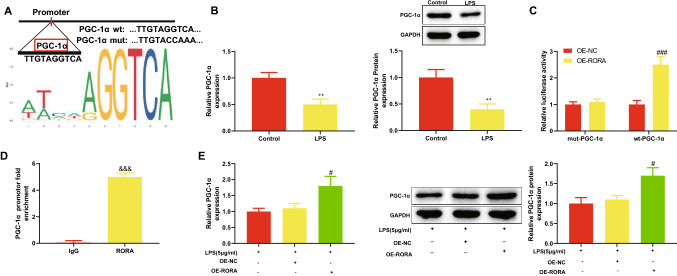
RORA binds PGC-1α promoter and promotes PGC-1α transcription. **A** Jaspar analyzed the relationship between RORA and PGC-1α; wild and mutant sequences of the binding site in PGC-1α promoter were synthesized for dual-luciferase reporter assay. **B** qRT-PCR and Western blotting were used to detect PGC-1α mRNA and protein in HK-2 cells before and after LPS treatment. Dual-luciferase reporter assay (**C**) and chromatin immunoprecipitation assay (**D**) were performed to verify the binding of RORA to PGC-1α promoter. **E** qRT-PCR and Western blotting were used to analyze the effect of OE-RORA transfection on the expression of PGC-1α mRNA and protein in LPS-treated HK-2 cells. Data were expressed as mean ± standard deviation and each experiment was repeated 3 times. **P < 0.01, compared with control group. ^#^*P* < 0.05 and ^###^*P* < 0.001, compared with LPS + OE-NC group. ^&&&^*P* < 0.001, compared with IgG group. RORA, retinoic acid receptor-related orphan receptor alpha; PGC-1α, peroxisome proliferator-activated receptor gamma coactivator 1 alpha; qRT-PCR, quantitative real-time polymerase chain reaction; LPS, lipopolysaccharide; OE-RORA, RORA overexpression vector; OE-NC, negative control vector

From the last result, we found overexpression of RORA reduced LPS-induced apoptosis of renal epithelial cells, but the relevant mechanism remained unclear. We used the Jaspar database (http://jaspar.genereg.net/) to predict the relationship between RORA and PGC-1α and found a RORA-binding site on PGC-1α promoter (Fig. 2A). First, we analyzed the expression of PGC-1α in HK-2 cells after LPS treatment. The results of qRT-PCR and Western blotting showed that LPS treatment significantly reduced the expression of PGC-1α mRNA and protein in HK-2 cells (Fig. 2B, **P < 0.01). Next, dual-luciferase reporter and ChIP assays were performed to confirm whether RORA could bind PGC-1α. HEK293T cells expressed more luciferase when transfected with wt-PGC-1α and OE-RORA (Fig. 2C, ^###^*P* < 0.001); the relative luciferase activity remained unchanged when cells were transfected with mut-PGC-1α and OE-RORA (Fig. 2C). In the ChIP assay, anti-RORA instead of anti-IgG significantly captured PGC-1α promoter (Fig. 2D, ^&&&^*P* < 0.001). To verify the regulation of RORA on the transcription of PGC-1α, we delivered OE-RORA or OE-NC into LPS-treated HK-2 cells and then detected the expression of PGC-1α using qRT-PCR and Western blotting. Compared with OE-NC transfection, OE-RORA transfection significantly promoted the expression levels of PGC-1α mRNA and protein in LPS-treated HK-2 cells (Fig. 2E, ^#^*P* < 0.05). The above results indicated that RORA promoted the transcription of PGC-1α by binding PGC-1α promoter.

### Overexpression of PGC-1α reduces LPS-induced apoptosis of renal epithelial cells

**Fig. 3:**
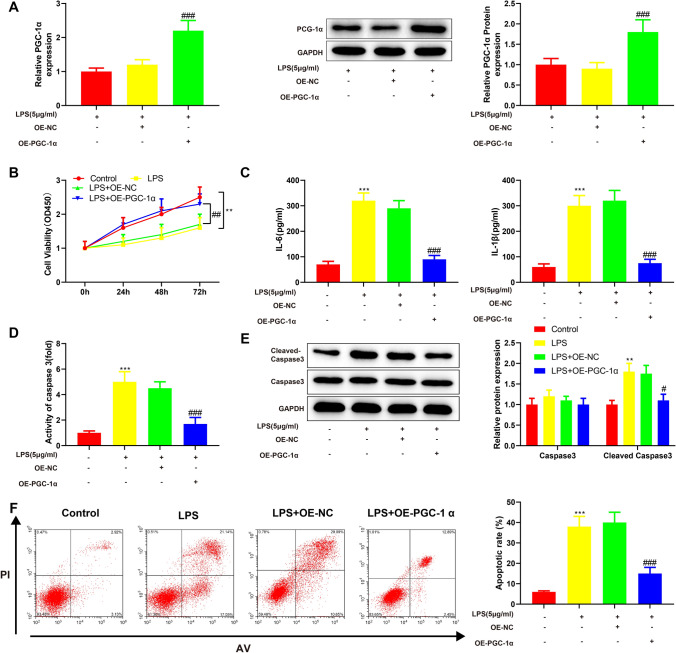
Overexpression of PGC-1α reduces LPS-induced apoptosis of renal epithelial cells. LPS-treated HK-2 cells were transfected with OE-PGC-1α or OE-NC. **A** qRT-PCR and Western blotting were used for analyzing the transfection efficiency of OE-PGC-1α. **B** CCK-8 was used for evaluating cell proliferation. ELISA was performed to detect the expression of IL-6 and IL-1β (**C**) and the activity of caspase-3 (**D**). **E** Western blotting was used to detect caspase-3 and cleaved caspase-3. **F** Flow cytometry was applied for analyzing cell apoptosis. Data were expressed as mean ± standard deviation and each experiment was repeated 3 times. **P < 0.01 and ***P < 0.001, compared with control group. ^#^*P* < 0.05, ^##^*P* < 0.01 and ^###^*P* < 0.001, compared with LPS + OE-NC group. PGC-1α, peroxisome proliferator-activated receptor gamma coactivator 1 alpha; LPS, lipopolysaccharide; OE-PGC-1α, PGC-1α overexpression vector; OE-NC, negative control vector; qRT-PCR, quantitative real-time polymerase chain reaction; CCK-8, cell counting kit-8; ELISA, enzyme-linked immunosorbent assay; IL, interleukin

From the above, PGC-1α was lowly expressed in HK-2 cells after LPS treatment. To investigate the function of PGC-1α in AKI, we delivered PGC-1α overexpression vector (OE-PGC-1α) or its negative control (OE-NC) into LPS-treated HK-2 cells. The results of qRT-PCR and Western blotting showed that OE-PGC-1α transfection significantly increased the expression levels of PGC-1α mRNA and protein in LPS-treated HK-2 cells (Fig. 3A, ^###^*P* < 0.001). We tested the viability of HK-2 cells using CCK-8 and found the proliferative ability of LPS-treated HK-2 cells was enhanced by OE-PGC-1α transfection compared with OE-NC transfection (Fig. 3B, ^##^ *P* < 0.01). ELISA was performed to detect the expression of IL-1β and IL-6 in cell supernatant and the activity of apoptosis factor caspase-3 in cells. Compared with OE-NC transfection, OE-PGC-1α transfection reduced the expression of IL-1β and IL-6 and the activity of caspase-3 (Fig. 3C, D, ^##^*P* < 0.01, ^###^*P* < 0.001). In addition, the Western blots showed there was a decrease in the expression of cleaved caspase-3 in LPS-treated HK-2 cells after OE-PGC-1α transfection (Fig. 3E, ^#^*P* < 0.05). The flow cytometry analysis showed that compared with OE-NC transfection, OE-PGC-1α transfection significantly inhibited the apoptosis of LPS-treated HK-2 cells (Fig. 3F, ^###^*P* < 0.001). Taken together, these data indicated that overexpression of PGC-1α reduced LPS-induced apoptosis of HK-2 cells.

### RORA alleviates LPS-induced renal epithelial cell apoptosis by promoting PGC-1α transcription

**Fig. 4:**
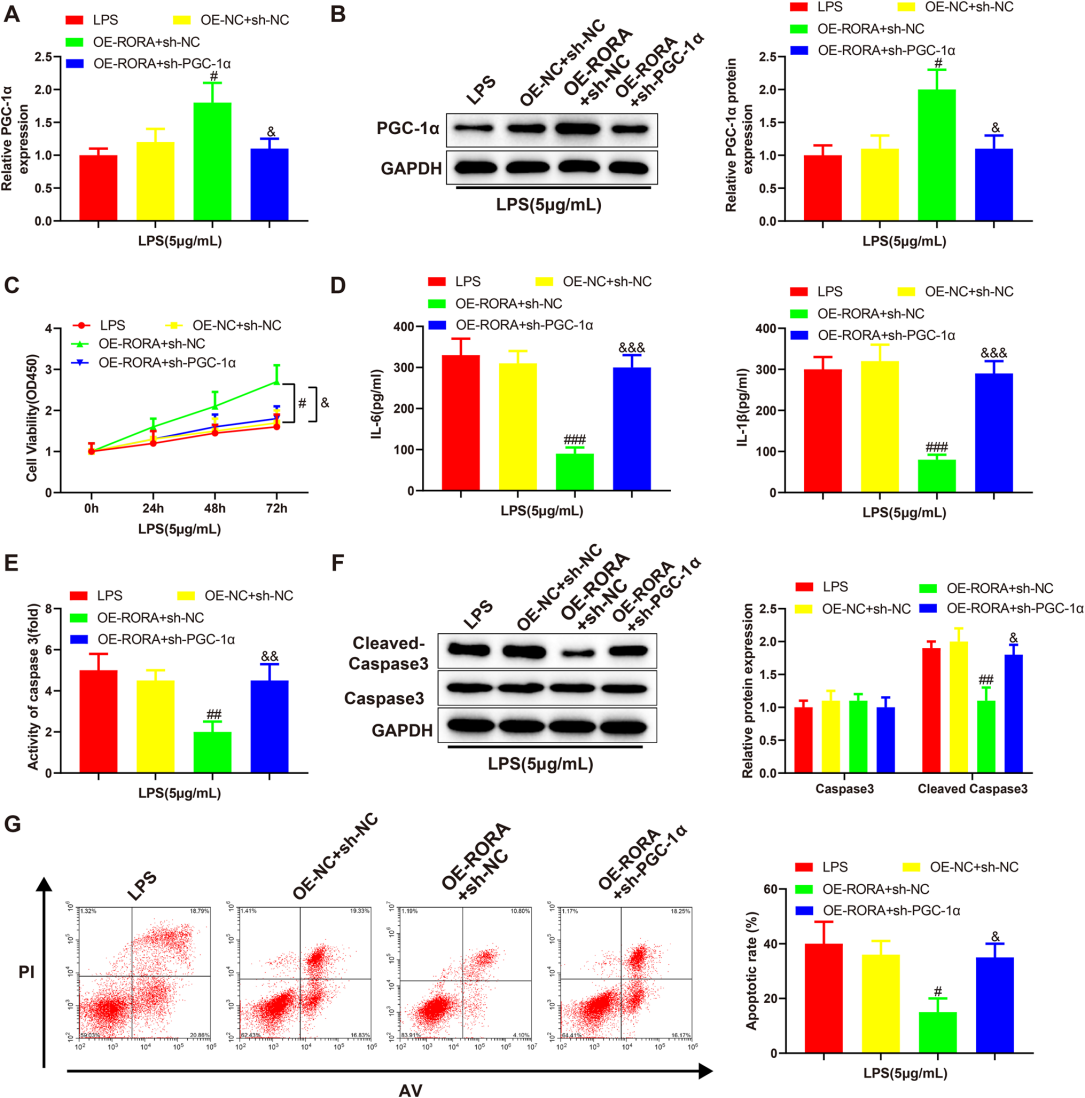
RORA alleviates LPS-induced renal epithelial cell apoptosis by promoting PGC-1α transcription. LPS-treated HK-2 cells were transfected with OE-RORA + sh-PGC-1α or OE-RORA + sh-NC. qRT-PCR (**A**) and Western blotting (**B**) were used to detect PGC-1α mRNA and protein. **C** CCK-8 was used for evaluating cell proliferation. ELISA was performed to detect the expression of IL-6 and IL-1β (**D**) and the activity of caspase-3 (**E**). **F** Western blotting was used to detect caspase-3 and cleaved caspase-3. **G** Flow cytometry was applied for analyzing cell apoptosis. Data were expressed as mean ± standard deviation and each experiment was repeated 3 times. ^#^*P* < 0.05, ^##^*P* < 0.01 and ^###^*P* < 0.001, compared with OE-NC + sh-NC group. ^&^*P* < 0.05, ^&&^*P* < 0.01 and ^&&&^*P* < 0.001, compared with OE-RORA + sh-NC group. RORA, retinoic acid receptor-related orphan receptor alpha; PGC-1α, peroxisome proliferator-activated receptor gamma coactivator 1 alpha; LPS, lipopolysaccharide; OE-RORA, RORA overexpression vector; sh-PGC-1α, PGC-1α inhibition vector; sh-NC, negative control vector; qRT-PCR, quantitative real-time polymerase chain reaction; CCK-8, cell counting kit-8; ELISA, enzyme-linked immunosorbent assay; IL, interleukin

In the above experiments, we found that overexpression of RORA or PGC-1α reduced LPS-induced apoptosis of HK-2 cells and that RORA could bind the promoter of PGC-1α. To confirm whether RORA reduced the apoptosis of LPS-treated HK-2 cells by promoting PGC-1α transcription, we delivered OE-RORA together with PGC-1α inhibition vector (sh-PGC-1α) or negative control vector (sh-NC) into LPS-treated HK-2 cells. These cells were accordingly divided into OE-RORA + sh-PGC-1α and OE-RORA + sh-NC groups. qRT-PCR and Western blotting were used to detect the expression levels of PGC-1α mRNA and protein. The results showed that OE-RORA transfection-induced enhancement of PGC-1α expression was eliminated in the presence of sh-PGC-1α (Fig. 4A, B, ^&^*P* < 0.01). Next, we explored the effect of OE-RORA and sh-PGC-1α co-transfection on LPS-induced apoptosis of renal epithelial cells. The results of CCK-8 assay showed that the proliferation of LPS-treated HK-2 cells was significantly inhibited in the OE-RORA + sh-PGC-1α group compared with the OE-RORA + sh-NC group (Fig. 4C, ^&^*P* < 0.01). The results of ELISA showed that the expression of IL-1β and IL-6 and the activity of caspase-3 were promoted in the OE-RORA + sh-PGC-1α group compared with the OE-RORA + sh-NC group (Fig. 4D, E, ^&&^*P* < 0.01, ^&&&^*P* < 0.001). The expression of cleaved caspase-3, showed by Western blots, was increased in the OE-RORA + sh-PGC-1α group compared with the OE-RORA + sh-NC group (Fig. 4F, ^#^*P* < 0.05). The flow cytometry analysis further indicated that the apoptosis of LPS-treated HK-2 cells was stimulated in the OE-RORA + sh-PGC-1α group compared with the OE-RORA + sh-NC group (Fig. 4G, ^&^*P* < 0.05). From this paragraph, RORA reduced the apoptosis of LPS-treated HK-2 cells by promoting PGC-1α transcription.

## Discussion

Biomarkers of AKI have been discovered and analyzed over the years. These molecules signify damage to the kidney and are suggested as adjunct diagnostics or targeted therapeutics to improve the early detection and clinical treatment of AKI [15]. LPS is a common inducer of AKI and causes renal dysfunction through mechanisms such as renal cell apoptosis, inflammatory immune responses and superoxide damage [16]. The authors used a LPS-induced cell model of AKI to study its pathogenesis and found RORA and PGC-1α played significant roles in renal epithelial cell apoptosis after AKI. The relationship between RORA and PGC-1α was analyzed using bioinformatics tools and confirmed using luciferase reporter and immunoprecipitation methods.

RORA was downregulated in HK-2 cells after LPS treatment. Overexpression of RORA stimulated the proliferative ability of LPS-treated HK-2 cells and reduced their production of inflammatory cytokines IL-6 and IL-1β. Moreover, overexpression of RORA decreased the activity of caspase-3 and alleviated the apoptosis of LPS-treated HK-2 cells. RORA constitutively activates transcription through recruitment of transcriptional co-activators and regulates metabolism, development and immunity in many tissues [17]. Kidney is a highly metabolic organ responsible for water and solute reabsorption and dysregulation of energy metabolism is seen in multiple types of AKI [18]. Inflammation is an important cause of AKI and the crosstalk between the kidney and immune system exists throughout the course of AKI [19]. Although the role of RORA in AKI remains largely unclear, RORA has been frequently reported to regulate metabolic activities or inflammatory responses in many other diseases. For example, overexpression of RORA reduced red blood cell infiltration and nasal mucosal injury in allergic rhinitis through blockade of Wnt/β-catenin signaling pathway [20]. Long noncoding RNA FGD5‑AS1 suppressed hypoxia‑induced oxidative stress and inhibited apoptosis of cardiomyocytes in acute myocardial infarction by upregulating the expression of RORA [21]. An agonist of RORA stimulated lipid metabolism by inducing microRNA-122 expression in a mouse model of nonalcoholic steatohepatitis, which subsequently reduced hepatic lipotoxicity and fibrosis [22].

Similar to RORA, PGC-1α was downregulated in HK-2 cells after LPS treatment. The proliferation of LPS-treated HK-2 cells was rescued by overexpression of RORA, while the secretion of inflammatory cytokines and the apoptosis of LPS-treated HK-2 cells were inhibited. More importantly, through in silico and in vitro experiments, we found RORA promoted the transcription of PGC-1α by binding PGC-1α promoter. Knockdown of PGC-1α offset the reduction in HK-2 cell injury caused by overexpression of RORA. PGC-1α is a versatile metabolic regulator expressed in many tissues; in the kidney, it maintains metabolic homeostasis and renal cell function by regulating fed-to-fasted energy transition as well as diurnal rhythm [23]. Considering the critical role of PGC-1α in regulating renal metabolism, many studies have been carried out to analyze the function of PGC-1α in AKI. For example, a deficiency of PGC-1α was observed in both human and experimental AKI, which was associated with exacerbation of tubular cell death and pro-inflammatory respons [24]. Nitrosporeusine A attenuated cecal ligation and puncture-induced septic AKI by reducing oxidative stress through upregulation of PGC-1α [25]. Overexpression of PGC-1α inhibited renal cell apoptosis and mitochondrial dysfunction in cisplatin-induced AKI through activation of TFEB-dependent mitophagy [26].

The findings of this study demonstrate that RORA reduces LPS-induced apoptosis of renal epithelial cells by promoting the transcription of PGC-1α. This study is the first to uncover the downstream target of RORA in regulating AKI and further confirms the nephroprotective effect of RORA. Nonetheless, the function of RORA needs further validation in animal models and clinical settings.
